# Diverse Fluoroquinolone Resistance Plasmids From Retail Meat *E. coli* in the United States

**DOI:** 10.3389/fmicb.2019.02826

**Published:** 2019-12-05

**Authors:** Gregory H. Tyson, Cong Li, Chih-Hao Hsu, Sonya Bodeis-Jones, Patrick F. McDermott

**Affiliations:** Office of Research, Center for Veterinary Medicine, U.S. Food and Drug Administration, Laurel, MD, United States

**Keywords:** PacBio, antimicrobial resistance, *Escherichia coli*, plasmids, fluoroquinolones

## Abstract

Fluoroquinolones are used to treat serious bacterial infections, including those caused by *Escherichia coli* and *Salmonella enterica*. The emergence of plasmid-mediated quinolone resistance (PMQR) represent a new challenge to the successful treatment of Gram-negative infections. As part of a long-term strategy to generate a reference database of closed plasmids from antimicrobial resistant foodborne bacteria, we performed long-read sequencing of 11 *E. coli* isolates from retail meats that were non-susceptible to ciprofloxacin. Each of the isolates had PMQR genes, including *qnrA1*, *qnrS1*, and *qnrB19*. The four *qnrB19* genes were carried on two distinct ColE-type plasmids among isolates from pork chop and ground turkey and were identical to plasmids previously identified in *Salmonella*. Seven other plasmids differed from any other sequences in GenBank and comprised IncF and IncR plasmids that ranged in size from 48 to 180 kb. These plasmids also contained different combinations of resistance genes, including those conferring resistance to beta-lactams, macrolides, sulfonamides, tetracycline, and heavy metals. Although relatively few isolates have PMQR genes, the identification of diverse plasmids in multiple retail meat sources suggests the potential for further spread of fluoroquinolone resistance, including through co-selection. These results highlight the value of long-read sequencing in characterizing antimicrobial resistance genes of public health concern.

## Introduction

Fluoroquinolones such as ciprofloxacin are critically important antimicrobials in human medicine. They are used to treat serious bacterial infections caused by Gram-negative and Gram-positive organisms (Parry and Threlfall, 2008; Camins et al., 2011). Thus, fluoroquinolone resistance is a public health issue that can lead to treatment failures and the use of alternative agents with greater side effects.

Fluoroquinolones are bactericidal antimicrobials that disrupt DNA replication in bacterial cells. Fluoroquinolone resistance is often mediated by mutations in the DNA gyrase and topoisomerase genes, with *gyrA* mutations being the most common mechanism in Gram-negative bacteria (Reyna et al., 1995). In recent years, plasmid-mediated quinolone resistance (PMQR) has become more frequent and can spread resistance through horizontal gene transmission. Known genes include *qnr*, *aac(6′)-Ib-cr*, *qepA*, and *oqxAB* (Ruiz et al., 2012). There are several types of *qnr* genes, including *qnrA*, *qnrB*, *qnrC*, *qnrD*, *qnrE*, *qnrS*, and *qnrVC*, which together have over one hundred named alleles (Ruiz, 2019). The presence of these genes differs by bacterial species, with *qnrA*, *qnrB*, and *qnrS* being most common among *Salmonella enterica* and *Escherichia coli* in the United States (Tyson et al., 2017b).

Although PMQR often results in only low-level fluoroquinolone resistance, this can then result in continued selection for *gyrA* mutants with even higher levels of resistance (Kim et al., 2009). Transmissible fluoroquinolone resistance is significant since it can lead to the rapid spread of resistance in bacterial species of public health importance. Cross-species and cross-genus transfer of resistance determinants is also possible. For example, *qnrB* genes are thought to have originated in *Citrobacter* spp. (Ribeiro et al., 2015), but have now been found in many pathogens, including *E. coli*, *S. enterica*, and *Klebsiella pneumoniae*, among others (Gay et al., 2006; Jacoby et al., 2006; Robicsek et al., 2006).

The National Antimicrobial Resistance Monitoring System (NARMS) is a One Health surveillance system in the United States that monitors antimicrobial resistance of foodborne pathogens from food animals, retail meats, and humans. Previous NARMS surveillance has found that the prevalence of PMQR genes in the food supply in the United States is low (McDermott et al., 2016), although ColE plasmids carrying *qnrB19* have been found in *Salmonella* from swine sources (Tyson et al., 2017a).

Plasmids containing multiple resistance genes can also co-select for resistance, as the use of one antimicrobial can select for resistance to additional drug classes (Vien le et al., 2012). Thus, it is important to identify and characterize resistance plasmids, particularly those conferring fluoroquinolone resistance.

In this paper, we report the use of long-read sequencing to characterize PMQR plasmids among *E. coli* isolated from retail meats in the United States, showing the diversity of mechanisms and potential co-selection.

## Materials and Methods

### Bacterial Strains

*Escherichia coli* strains were isolated from routine NARMS retail meat sampling from 13 states in 2015–2017 (NARMS, 2017). Antimicrobial susceptibility testing was performed per CLSI methods, with ciprofloxacin non-susceptibility defined per CLSI M100-S29 as MICs ≥ 0.125 μg/mL (CLSI, 2019).

### Sequencing and Assembly

Genomic DNA was extracted with DNeasy Blood and Tissue Kits (Qiagen, Valencia, CA, United States) per the manufacturer’s instructions. Whole-genome sequencing was performed on the Pacific Biosciences (PacBio) Sequel Sequencer, as previously described (Tyson et al., 2019). Continuous long reads were assembled by the PacBio Hierarchical Genome Assembly Process (HGAP4.8) program. Assembly of the *qnrB19* plasmids was done using CLC Genomics Workbench version 10.0.

### Annotation and Sequencing Analysis

The closed genomes were annotated by the Prokaryotic Genome Annotation Pipeline (PGAP) version 4.8 (Tatusova et al., 2016). Resistance genes were identified by the NCBI Pathogen Detection Pipeline by AMRFinder (Feldgarden et al., 2019). BLASTn was also used to compare plasmids identified with those in GenBank. Plasmid typing was determined by PlasmidFinder, comparing with the database to known plasmid types with 95% sequence identity and 60% sequence length (Carattoli et al., 2014). Multi-locus sequence typing (MLST) was done with assembled sequences using MLST 2.0 (Larsen et al., 2012), using the *E. coli* scheme previously described (Wirth et al., 2006). Sequences were submitted to GenBank, with BioSamples in Table 1 and plasmid nucleotide accession numbers listed in Table 2.

**TABLE 1:** Metadata for isolates with PMQR genes.

**Isolate ID**	**Source**	**Year**	**State**	**ST**	**CIP MIC (μg/mL)**	**PMQR mechanism**	**BioSample**
N55972	Pork chop	2015	GA	10	0.25	*qnrB19*	SAMN12698087
N56338	Pork chop	2015	GA	1079	0.5	*qnrB19*	SAMN12698088
N56639	Ground beef	2015	GA	5180^∗^	0.5	*qnrA1*	SAMN12698089
N62675	Ground turkey	2015	GA	398	0.25	*qnrS1*	SAMN12698090
N16EC0140	Pork chop	2016	OR	13	0.25	*qnrB19*	SAMN12698091
N16EC0879	Ground turkey	2016	TN	58	0.25	*qnrS1*	SAMN12698092
N17EC0211	Ground turkey	2017	IA	540	0.12	*qnrA1*	SAMN10221061
N17EC0320	Ground turkey	2017	OR	540	0.12	*qnrA1*	SAMN10221115
N17EC0326	Ground turkey	2017	OR	10	0.25	*qnrB19*	SAMN10221118
N17EC0616	Chicken leg	2017	CO	1485	0.12	*qnrS1*	SAMN10221255
N17EC1164	Pork chop	2017	TX	2207	0.25	*qnrS1*	SAMN10221523

*^∗^Closest ST type to that of this isolate, as its *fumC* allele differs from that of ST5180.*

**TABLE 2:** Characteristics of PMQR plasmids.

**Isolate ID**	**Plasmid type**	**Plasmid length**	**Accession**	**Plasmid resistance genes**
P3_N55972	ColE	3,071	CP043760	*qnrB19*
P2_N56338	ColE	3,071	CP043756	*qnrB19*
P1_N56639	IncR	48,263	CP043753	*qnrA1 aadA2 bla*_CARB–__1_ *mphA floR sul1 (3x) tetA dfrA1*
P1_N62675	IncR	81,916	CP043751	*qnrS1 aadA2 dfrA12 sul3 tetA*
P2_N16EC0140	ColE	3,071	CP043749	*qnrB19*
P1_N16EC0879	IncF	138,918	CP043745	*qnrS1 bla*_CTX–M–__55_ *tetA aac(3)-IId*
P1_N17EC0211	IncF	125,644	CP043743	*qnrA1 bla*_CARB–__2_ *aadA2 (2x) dfrA12 ant(3″)-Ia cmlA1 sul1 sul3 tetA*
P2_N17EC0320	IncF	126,972	CP043741	*qnrA1 bla*_CARB–__2_ *aadA2 (2x) dfrA12 ant(3″)-Ia cmlA1 sul1 sul3 tetA*
P4_N17EC0326	ColE	2,699	ROAP02000006	*qnrB19*
P1_N17EC0616	IncF	179,651	CP043737	*qnrS1 bla*_TEM–__1__B_ *strA strB sul2 dfrA14 tetA*
P1_N17EC1164	IncR	101,987	CP043734	*qnrS1 bla*_TEM–__1__B_ *tetA sul3 ant(3″)-Ia cmlA1 aadA2 dfrA12 tet(M)*

## Results

From 2015 to 2017, NARMS recovered 3,267 *E. coli* isolates from retail meat sampling. We performed short-read sequencing on approximately 1,500 of these isolates to identify resistance mechanisms. Most isolates with fluoroquinolone resistance mechanisms had *gyrA* mutations, comprising 42 isolates. Another 11 isolates lacked gyrase mutations but carried PMQR genes, including *qnrA1*, *qnrS1*, and *qnrB19*. Isolates with these genes were from a variety of sources, including retail chicken, turkey, beef, and pork (Table 1). There were no isolates with both PMQR genes and *gyrA* mutations.

To further characterize the isolates with PMQR genes, we performed long-read sequencing using Pacific Biosciences technology. From this sequencing, we obtained closed, circular chromosomes and plasmids from each isolate.

Four of the isolates possessed *qnrB19* genes, which were found on small plasmids as expected. Long-read sequencing is not optimal for plasmids under 10 kb, so plasmids were closed using short-read sequencing data. We identified two distinct ColE-type plasmids of approximately 3 kb (Table 2) containing these genes. Interestingly, one of the isolates was from ground turkey, and had a different plasmid from the other three isolates, which were from pork chop. The four isolates were genetically distinct, comprising three different *E. coli* sequence types (STs) (Table 1). The two isolates that were ST10 were from different sources and not within 50 single-nucleotide polymorphisms (SNPs) of each other in the NCBI Isolates Browser (Feldgarden et al., 2019). The two plasmids we found were identical to those identified in swine sources of *Salmonella* in the United States (Tyson et al., 2017a) and have also been found in *E. coli* and *Salmonella* in South America (Pallecchi et al., 2010).

**FIGURE 1:**
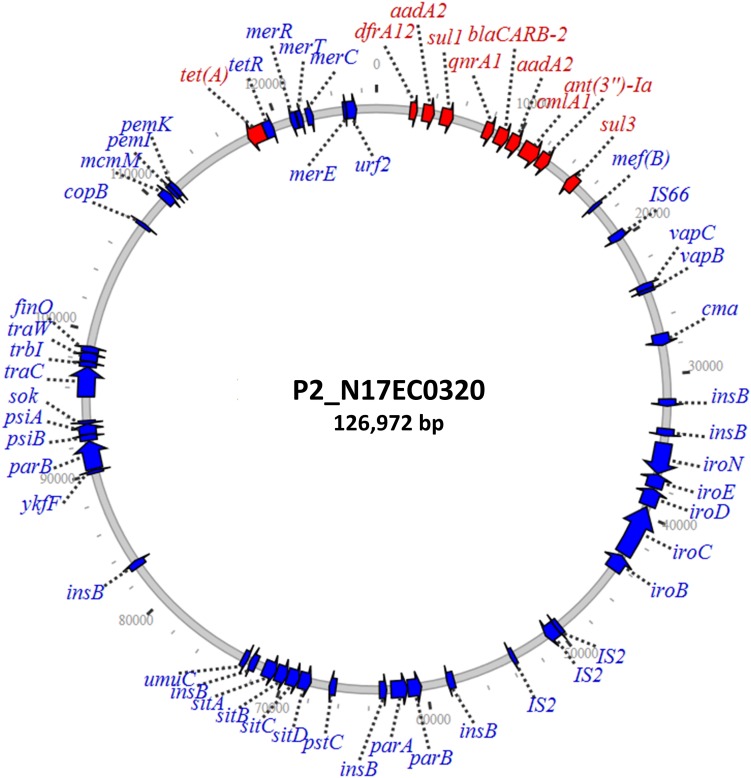
IncF plasmid containing *qnrA1* and other resistance genes. Resistance genes are depicted in red, with additional annotated genes depicted in blue.

Three isolates had plasmids containing *qnrA* genes, with two isolates from ground turkey and one from ground beef. The ground turkey isolates were both identified as ST540 from *in silico* MLST (Larsen et al., 2012), and were 15 SNPs away from each other according to the NCBI Isolates Browser (Feldgarden et al., 2019). These two isolates had nearly identical PMQR plasmids of approximately 126 kb each, indicating likely clonal spread of this strain and its plasmid. These IncF plasmids had limited homology to known plasmids, and contained genes conferring resistance to beta-lactams, aminoglycosides, phenicols, sulfonamides, and tetracycline (Table 2). A graphical representation of one of these plasmids is shown in Figure 1. Interestingly, this plasmid also contains the *iroN*, *iroBCDE*, and *sitABCD* genes, which are involved in iron uptake and may contribute to virulence (Russo et al., 2002; Sabri et al., 2008). Furthermore, the plasmid contains the *copB* gene, which exports copper and confers copper resistance (Vollmecke et al., 2012), as well as the *mer* operon, which encodes mercury resistance (Hamlett et al., 1992). Thus, the presence of copper or mercury could co-select for fluoroquinolone resistance.

**FIGURE 2:**
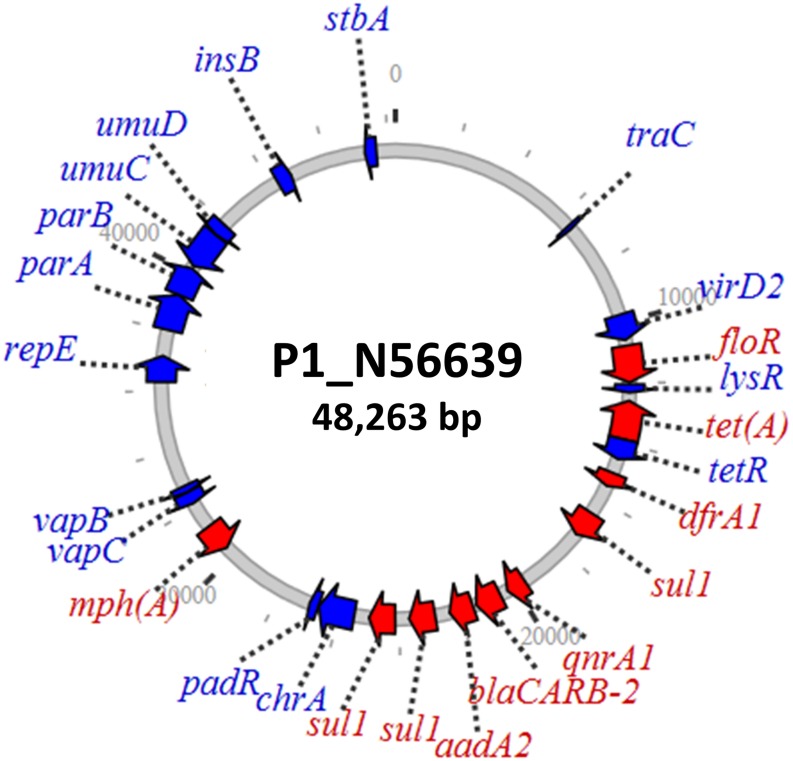
IncR plasmid containing *qnrA1* with various additional resistance genes.

The *qnrA* plasmid from the ground beef isolate comprised a 48-kb IncR replicon (Figure 2) with limited similarity to known plasmids. This plasmid had additional genes conferring resistance to beta-lactams, macrolides, phenicols, and sulfonamides. Since fluoroquinolones, beta-lactams, and macrolides are some of the most important antimicrobials used to treat serious Gram-negative infections, potential transfer of this plasmid to other pathogens could compromise the effectiveness of multiple potential therapies. The plasmid also contained the *vapBC* toxin–antitoxin system, which plays a role in greater plasmid stability (Winther and Gerdes, 2012) that may help with plasmid persistence. This toxin–antitoxin system was also present in the IncF plasmids containing *qnrA*.

**FIGURE 3:**
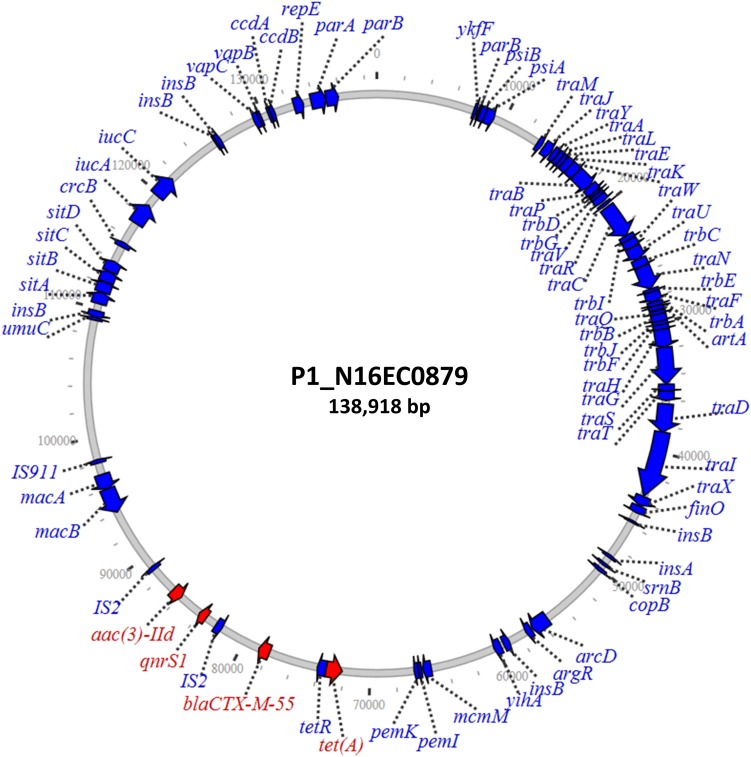
Large IncF plasmid containing *qnrS1* along with various additional resistance genes.

Four isolates had *qnrS* on unique IncF and IncR plasmids, with sizes from 81 to 180 kb (Table 2). These four isolates included those from retail pork, chicken, and turkey. One of these was an IncF plasmid from N16EC0879 that also had *bla*_CTX–M–__55_, *tetA*, and *aac(3)-IId* (Figure 3). The presence of *bla*_CTX–M–__55_ is noteworthy, as this is an extended-spectrum beta-lactamase gene that confers resistance to cephalosporins. Since cephalosporins such as ceftriaxone are used in human medicine, the combined transfer of both *bla*_CTX–M–__55_ and *qnrS1* on one plasmid could compromise therapy to multiple drug classes. While all four resistance genes were within a 15-kb fragment on the plasmid, only *qnrS1* and *aac(3)-IId* were on an insertion sequence together, an IS2 element. This plasmid also contained the iron uptake genes *sitABCD* and *iucAC* (Gordon et al., 2017), in addition to *macAB*, which may contribute to virulence (Yamanaka et al., 2008; Turlin et al., 2014). This plasmid also had the *copB* copper resistance gene, as described in the IncF plasmids with *qnrA*. The two isolates with *qnrS* on IncR plasmids also had the *sil* operon genes, which confer silver resistance (Table 2; Andrade et al., 2018).

Only a subset of the PMQR plasmids had identifiable conjugal transfer genes, such as those in the *tra* locus (Ilangovan et al., 2015). In fact, only the IncF plasmids had known conjugal transfer genes, indicating that the other plasmids may not be self-transmissible, or may require helper plasmids for successful spread through conjugation.

## Discussion

We report detailed sequence data on fluoroquinolone non-susceptible *E. coli* from retail meats. This work shows the value of long-read sequencing in *de novo* characterization of AMR plasmids. Using only short-read sequencing data makes it difficult to accurately identify plasmids or fully characterize them (George et al., 2017). Using short-read sequencing data alone we have accurately identified resistance genes in *E. coli* (Tyson et al., 2015), but not which are co-located on plasmids. In addition, assemblies from short-read data have difficulty identifying multiple copies of the same gene. For instance, some plasmids in this study had multiple copies of *aadA* and *sul* genes, but short-read data assemblies typically only identify single copies of resistance genes (Xia et al., 2017; Su et al., 2019). Understanding the full plasmid structure also helps uncover potential co-selection, including to heavy metals and other biocides. These details are important in assessing the nature of resistant microbial hazards in food and other sources. Fluoroquinolone use is relatively low in food animal production in the United States, but most PMQR plasmids also had genes conferring resistance to tetracycline, which is the highest selling antimicrobial for food animals in the United States (FDA, 2018). This means that tetracycline use in food animals could result in continued selection for fluoroquinolone resistance in *E. coli*.

Interestingly, *E. coli* from all retail meat food types had PMQR genes. This contrasts with prior findings of swine as the major contributor to PMQR in retail meat *Salmonella* (Tyson et al., 2017a). Most plasmids in this study had minimal homology to known plasmids. This demonstrates the importance of increased sequencing of plasmids even in well-studied bacteria such as *E. coli*, since completely new plasmids are still being discovered.

One interesting finding was that some *E. coli* had the same *qnrB19* plasmids as those in *Salmonella* from swine and retail pork from NARMS sampling (Tyson et al., 2017a). This reflects a likely transmission of plasmids between *E. coli* and *Salmonella*, including in non-swine sources. As observed in *Salmonella*, these isolates were diverse, indicating little serotype-specific barriers to transmission. These plasmids are small and likely not self-transmissible due to the lack of conjugation genes; each isolate had at least one additional plasmid, indicating a potential method for *qnrB19* plasmid transmission.

Bacteria from this study were generic *E. coli* unlikely to cause foodborne disease in humans, so the direct risk of these bacteria impacting human health or treatment is low. Furthermore, none of the isolates were within 50 SNPs of any human isolates in the NCBI Isolates Browser. Nevertheless, as a source of resistance of human health concern, these bacteria could transfer resistance plasmids to pathogenic *E. coli* or to other genera. The tracking and reporting of PMQR in these bacteria is essential for a One Health strategy to identify emerging public health threats, and is enhanced by long-read sequencing for *de novo* characterization of novel plasmids.

## Data Availability Statement

The datasets generated for this study can be found in the NCBI BioSamples SAMN12587179, SAMN12587180, SAMN12587181, SAMN12587182, SAMN12587183, SAMN12587184, SAMN102 21061, SAMN10221115, SAMN10221118, SAMN10221255, and SAMN10221523.

## Author Contributions

GT conceived and coordinated the study. GT and PM wrote the manuscript. CL performed the sequencing and did the sequencing analysis. C-HH did the sequencing analysis and made the figures. SB-J performed the antimicrobial susceptibility testing. All authors contributed to finalizing the manuscript.

## Disclaimer

The views expressed in this article are those of the authors and do not necessarily reflect the official policy of the Department of Health and Human Services, the U.S. Food and Drug Administration, or the U.S. Government. Reference to any commercial materials, equipment, or process does not in any way constitute approval, endorsement, or recommendation by the U.S. Food and Drug Administration.

## Conflict of Interest

The authors declare that the research was conducted in the absence of any commercial or financial relationships that could be construed as a potential conflict of interest.
